# OD (order–disorder) interpretation and diffuse scattering analysis of an organic polytype with allotwin character: a detailed how-to

**DOI:** 10.1107/S205252062500914X

**Published:** 2025-11-11

**Authors:** David Fröschl, Nicolas Kratena, Berthold Stöger, Alexandr Virovets, Tobias Wolflehner

**Affiliations:** a Institute of Applied Synthetic Chemistry, TU Wien, Getreidemarkt 9, 1060Vienna, Austria; b X-Ray Centre, TU Wien, Getreidemarkt 9, 1060Vienna, Austria; c Institute of Inorganic and Analytical Chemistry Goethe Universitaet Frankfurt,Max-von-Laue-Str. 7 60438Frankfurt Germany; d Institute of Chemical Technologies and Analytics, TU Wien, Getreidemarkt 9, 1060Vienna, Austria; Academy of Sciences of the Czech Republic, Czechia

**Keywords:** order–disorder theory, diffuse scattering, polytypes, twinning, allotwinning

## Abstract

2,3-Dihydroxy-1,3,4-trimethyl-6-oxo-1,4-cyclohexadiene-1-carboxylic acid crystallizes in an order–disorder (OD) structure with a high stacking fault probability. The diffraction pattern features diffuse scattering and broad peaks, which can be attributed to fragments of two polytypes of a maximum degree of order (MDO). Additional weak peaks indicate existence of a non-MDO polytype. A detailed step-by-step instruction is given on how to develop an OD description and interpret the diffuse scattering using a stochastic growth model.

## Abstract 2

The complex stacking disorder of 2,3-dihydroxy-1,3,4-trimethyl-6-oxo-1,4-cyclohexadiene-1-carboxylic is analyzed by applying the order–disorder theory and its diffuse scattering is interpreted using a growth model.

## 1. Introduction

Polytypes are structures that crystallize with identical (up to minor desymmetrization) layers arranged in different ways (Aksenov *et al.*, 2023 ▸). Polytypism is a multifarious phenomenon leading to interesting crystallographic challenges such as twinning (Nespolo & Ferraris, 2004 ▸), antiphase domains (Wondratschek & Jeitschko, 1976 ▸), allotwins (Nespolo *et al.*, 1999 ▸) and diffuse scattering (Welberry, 2010 ▸). Often, these appear in the same crystal and may not be separable.

We encountered an example of a such a complex crystallization behavior during a synthetic campaign towards 3,5-dimethylorselinic acid (DMOA)-derived meroterpenoid natural products. During the attempted alkaline hydrolysis of DMOA methyl ester (Powers *et al.*, 2019 ▸), a concomitant spontaneous oxidation towards the dearomatized dienone 2,3-dihydroxy-1,3,4-trimethyl-6-oxo-1,4-cyclohexadiene-1-carboxylic acid **1** was observed (Scheme 1).
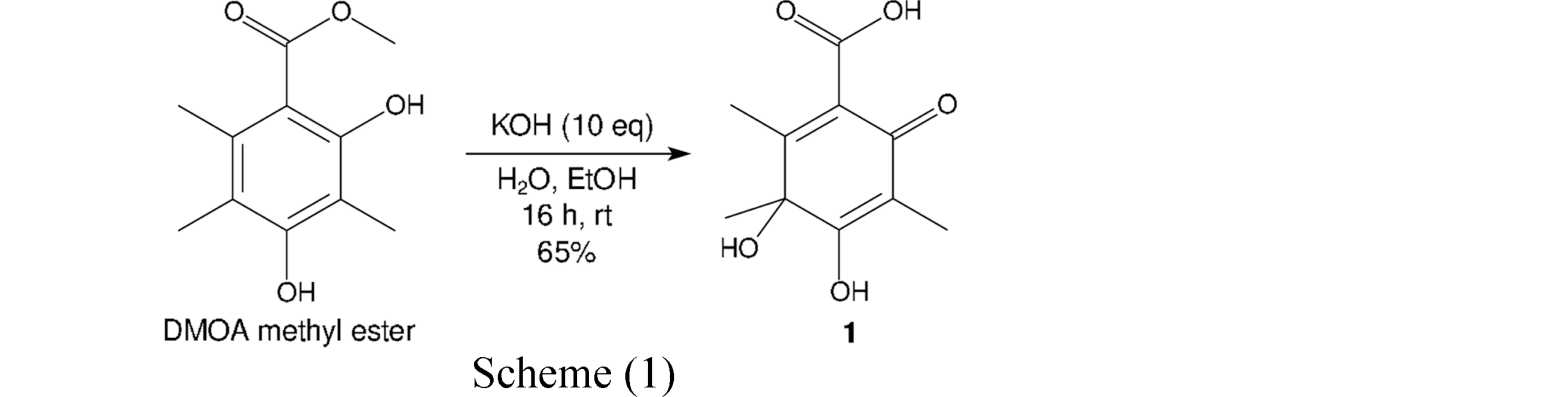


Presumably, the oxidative event takes place by nucleophilic attack of a phenolate with molecular oxygen. On routine structural characterization of **1** by single-crystal diffraction, we observed distinct diffuse scattering and broad peaks at positions that indicated fragments of distinct polytypes, *i.e.* an intermediate between a disordered structure and an allotwin. The phenomenon was systematic and observed for different crystals isolated from the same crystallization experiment.

The order–disorder (OD) theory (Dornberger-Schiff & Grell-Niemann, 1961 ▸) was created in the 1950s and further developed in the second half of the 20th century to explain the common occurrence of polytypism in all known classes of crystalline matter. It is based on the limited range of inter­atomic interactions. OD polytypes are an often observed class of polytypes, where layers can connect in different ways, nevertheless forming geometrically equivalent layer pairs. This means that, in theory, an infinite number of ordered polytypes or disordered stacking arrangements may form, which are all locally equivalent and form an OD family.

Herein, we will give a step-by-step introduction on how to develop an OD model based on the structure of **1**. For high-quality diffuse scattering reconstructions, we collected data of a selected crystal at the P24 beamline of the PETRA III synchrotron at DESY. A semi-quantitative interpretation of the diffraction pattern is given using growth models (Welberry, 2010 ▸). §3.6 and §3.11 contain detailed derivations, which may be skipped by the reader interested mainly in practical application of OD theory and growth models.

## 2. Experimental

### 2.1. Synthesis

The precursor DMOA methyl ester was obtained as per the published procedure by Porco *et al.* (2019 ▸).

Procedure: 50 mg of DMOA methyl ester (0.237 mmol, 1 equivalent) were dissolved in ethanol (1 ml) and stirred at room temperature. At the same time, 134 mg of KOH (2.38 mmol, 10 equivalents) were dissolved in deionized water (0.2 ml) and then added to the solution of the starting material. As soon as the base was added, the color of the solution changed from yellow to light red, during the next hour of stirring the solution turned dark red. The solution was stirred at room temperature for 20 h. Work up: the reddish-black solution was made acidic through addition of 3 M HCl (2 ml). The reaction mixture was extracted with ethyl acetate (4×), dried over magnesium sulfate and concentrated *in vacuo*. Purification: the product was purified through column chromatography (DCM/MeOH = 20/1 and 0.5% acetic acid). Yield: 33 mg (0.155 mmol, 65% of theory).

Single crystals were grown by dissolving 10 mg in a 1:1:1 mixture of acetone, ethanol and water and evaporating slowly through a septum with needle over 14 days.

### 2.2. Single-crystal diffraction

**Figure 1:**
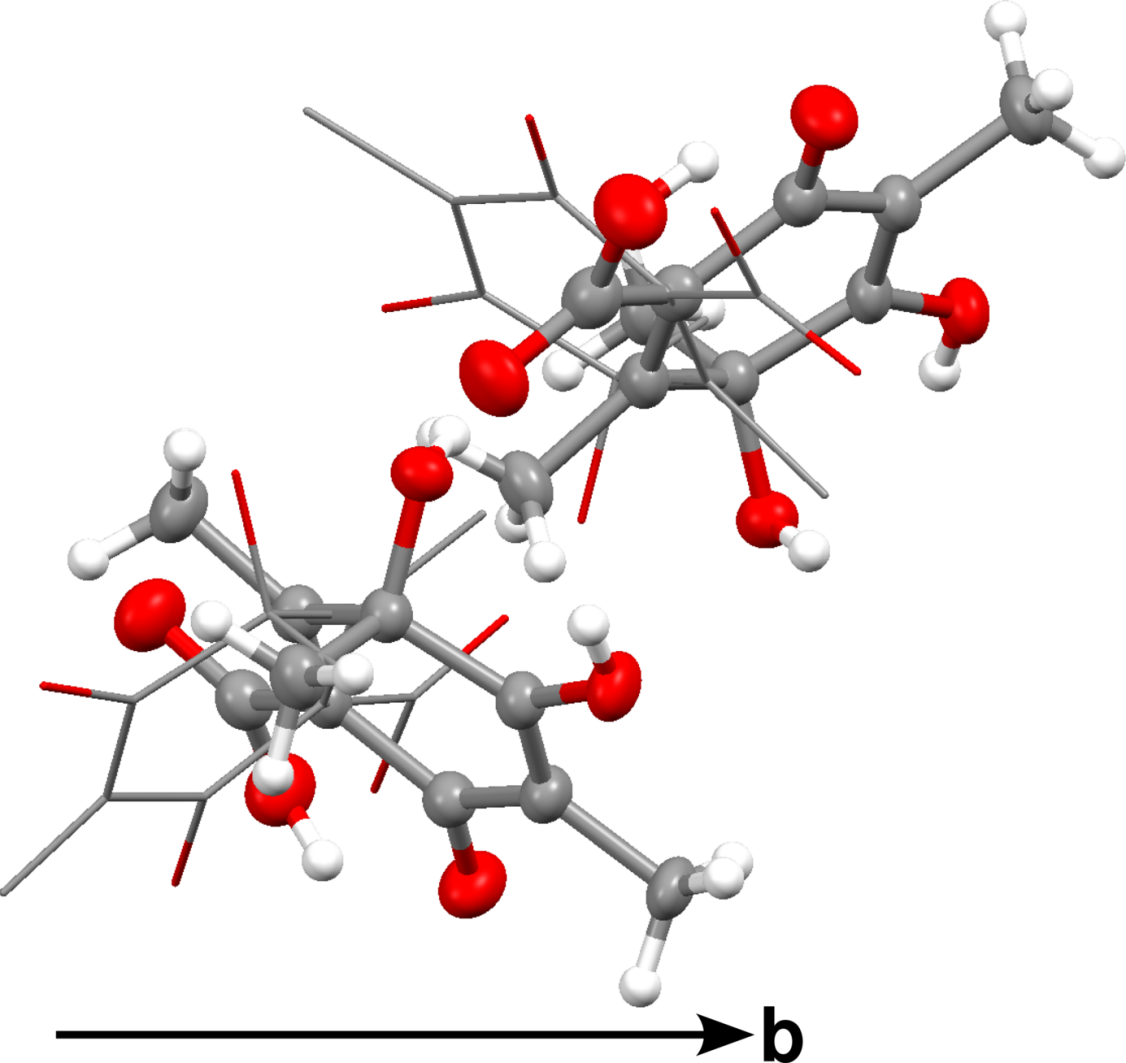
Asymmetric unit of the refined orthorhombic polytype viewed along [100]. Atoms are represented by red (O) and gray (C) ellipsoids drawn at the 50% probability levels; H atoms by white spheres of arbitrary radius. The phantom molecules are shown as wireframe.

Intensity data of a single crystal of **1** was collected at the high-flux P24 beamline of the PETRA III synchrotron using λ = 0.56 Å (*E* = 22.14 keV) radiation. Two 360° φ-scans with 0.2° frame width were collected. Data were converted into ESPERANTO format and processed with the *CrysAlis* software (Rigaku Oxford Diffraction, 2022 ▸). As discussed below, diffraction data consisted of reflections that could be attributed to multiple polytypes. The major polytype was orthorhombic and reflection intensities of other domains were omitted during structure refinement. The structure was solved with the dual-space approach implemented in *SHELXT* (Sheldrick, 2015*b* ▸) and refined against |*F*|^2^ using *SHELXL* (Sheldrick, 2015*a* ▸). The errors owing to ignoring reflections of alternative polytypes produce alternative orientations of the molecules in the difference Fourier maps, sometimes designated as ‘phantom molecules’. For each atom one major alternative position could be located in the difference Fourier map and the structure was refined as an overlay of two orientations of each molecule (Fig. 1 ▸). The displacement parameters of both orientations were constrained to be equal. Additional electron density peaks are attributed to even more alternative orientations, which were however too weak for inclusion in the refinements. Since the occupancy of the second orientation was only *ca* 11%, some atomic positions were ill-determined and distance restraints were implemented to achieve a reasonable geometry. The hydroxyl and carboxyl H atoms of the major orientation were refined with distance restraints, those of the minor orientation were omitted. More details on data collection and structure refinement are compiled in Table 1 ▸. Molecular graphics were prepared using the *Mercury* (Macrae *et al.*, 2020 ▸) software.

## 3. Results and discussion

### 3.1. Crystal chemistry

**Figure 2:**
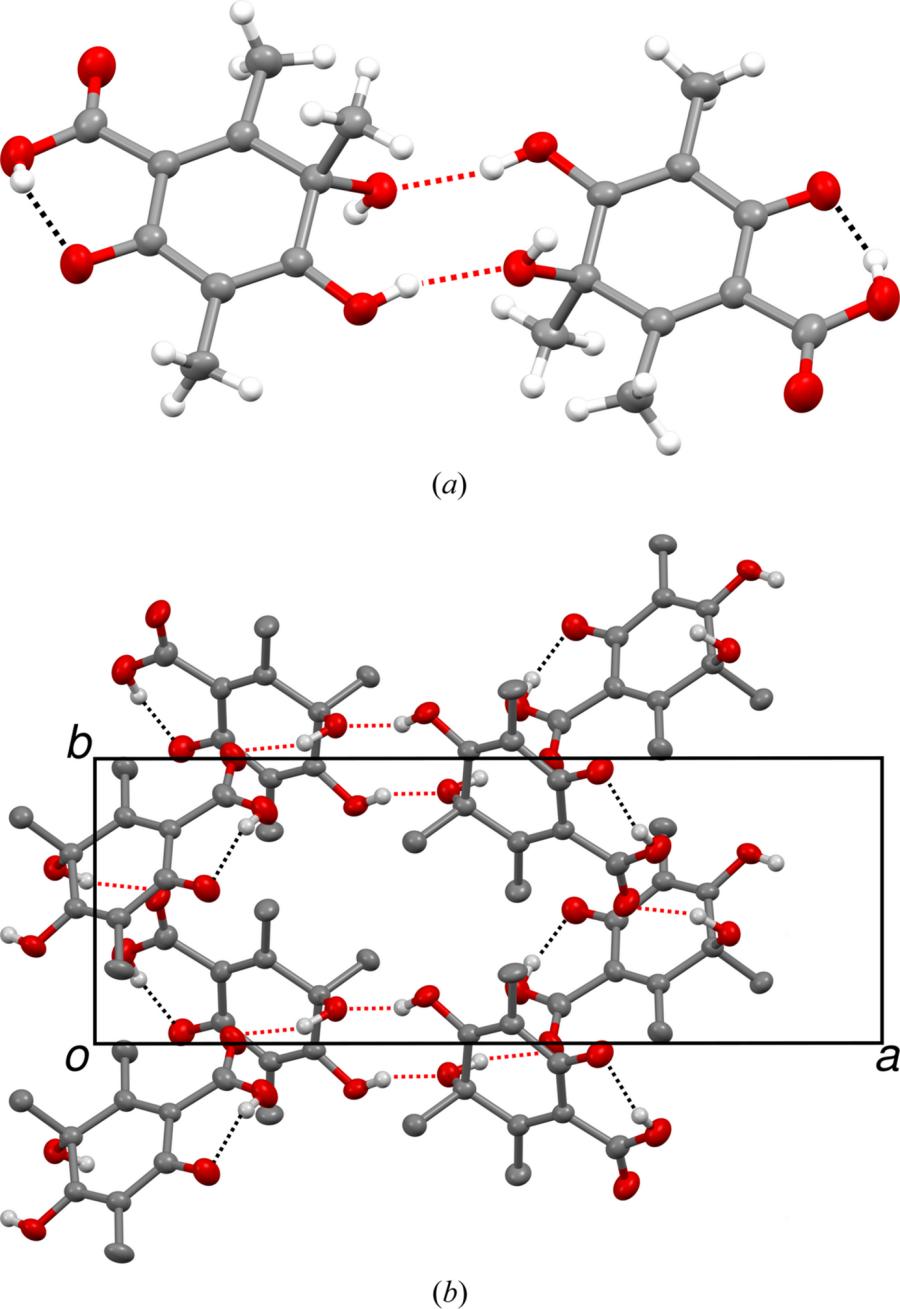
(*a*) Pair of **1** molecules connected by hydrogen bonds and (*b*) diperiodic hydrogen-bonding network extending parallel to the (001) plane. Atoms as in Fig. 1 ▸. Inter- and intramolecular hydrogen bonds are represented by red and black dashed lines, respectively.

The crystal chemistry of **1** will only be sketched briefly based on the major *Pna*2_1_ polytype. Molecule **1** [Fig. 2 ▸(*a*)] possesses three hydrogen bond donors. The carboxylic acid group forms an intramolecular hydrogen bond to the oxo group. Pairs of molecules related by pseudo-inversion symmetry are connected by intermolecular hydroxyl-to-hydroxyl bonds [see Fig. 2 ▸(*a*)]. Note that the two molecules are not equivalent according to the *Pna*2_1_ symmetry (hence *Z*′ = 2), which will play a crucial role in the description of the disorder. The remaining hydroxyl group forms an intermolecular bond to a carboxyl group, resulting in infinite sheets extending parallel to the (001) plane [Fig. 2 ▸(*b*)].

### 3.2. Deriving the OD layers

Even though application of OD theory is extremely useful from a descriptive but also a predictive point of view, its is not widely used. We attribute this to a lack of awareness and a perceived impenetrability. Therefore, here we give step-by-step instructions of how to derive the OD model of **1**.

An OD model should always be based on experimental evidence (for example twinning or diffuse scattering). For **1** there were four characteristic signs of stacking disorder.

(i) Pronounced one-dimensional diffuse scattering in c* direction.

(ii) ‘Phantom molecules’ of alternative stacking arrangements.

(iii) Crystallographically independent molecules (*Z*′ = 2) with systematic orientation relations.

(iv) Additional diffraction peaks of alternative polytypes.

None of these prove the OD character of the structure [see Schöbinger & Stöger (2024 ▸) for a counter-example], but they are a good indication.

The first step in an OD interpretation is identification of the OD layers, their (idealized) symmetry, and the operations relating layers. These operations need not be symmetry operations of the space group. They are *partial (symmetry) operations* (POs), because they apply only to distinct layers. The layers in **1** should be parallel to (001) in accordance with the observed diffuse scattering. Apart from the translations in the (001) plane, only the *a*_[010]_ glide reflection of the *Pna*2_1_ space group (and the compositions with the translations) maps potential layers parallel to (001) onto themselves. This however means that the symmetry groups of the OD layers must be supergroups of *p*1*a*1, where we use the notation of the *International Tables for Crystallography*, Vol. E (Kopsky & Litvin, 2006 ▸), which assumes layers extending parallel to the (001) plane.

**Figure 3:**
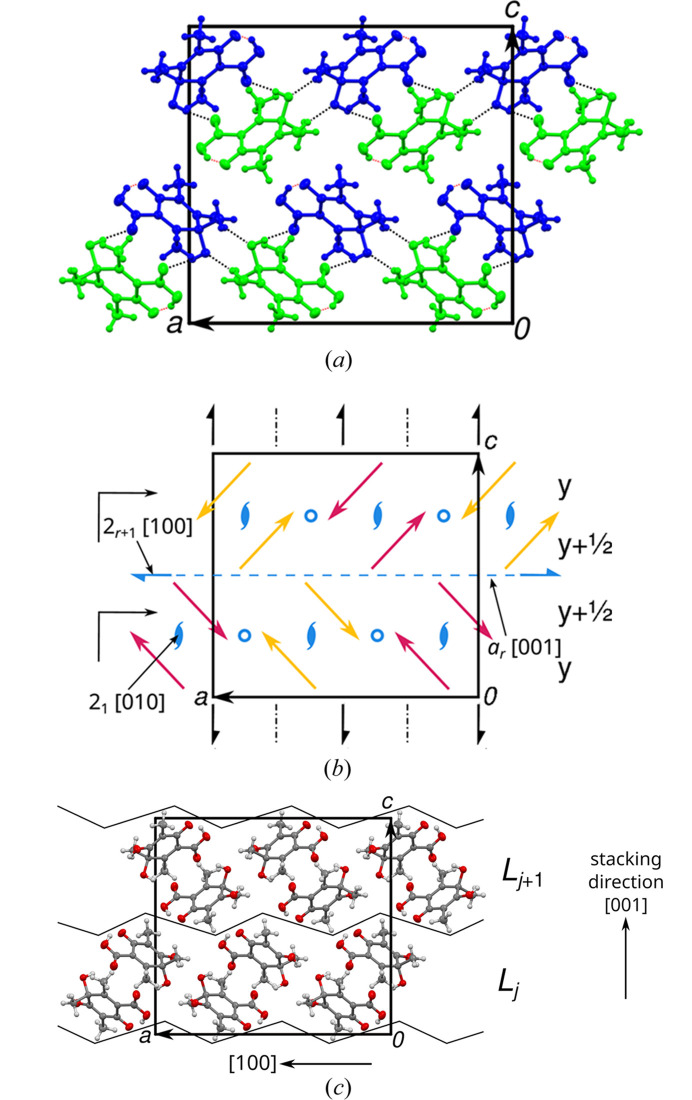
The *Pna*2_1_ polytype of **1** viewed down [010] with (*a*) molecules colored according to symmetry equivalence, (*b*) molecules represented by red (pointing toward the reader) and yellow (pointing away) arrows and (*c*) layer interfaces indicated by a black polygonal chain. In (*b*) symmetry elements of the space group (black) and non-space group symmetry elements (blue) are given by the common symbols. Additional translation along [010] is given to the right. Inter- and intramolecular hydrogen bonds are represented in (*a*) by black and red dashed lines, respectively. The geometric elements discussed in the text are marked by their printed symbol in (*b*). Atom colors and ellipsoids in (*c*) as in Fig. 2 ▸.

In the refined *Pna*2_1_ polytype, there are two non-equivalent molecules [marked by distinct colors in Fig. 3 ▸(*a*)]. Note that the stacking direction [001] in Fig. 3 ▸ and in the two-dimensional reciprocal space reconstructions below is pointing up, as is common in the OD literature and which is implied by the expression ‘layer stacking’. Since the molecules are ‘asymmetric’, or more precisely only symmetric by the trivial point group 1 (*C*_1_ after Schönfließ), any additional layer symmetry element must be located between molecules. In Fig. 3 ▸(*b*) a simplified view is given by replacing the molecules with arrows pointing from the 6-oxy group to the opposite 3-hydroxy group, whereby red and yellow colors give the orientation with respect to the viewing direction [010]. The symmetry elements of the *Pna*2_1_ space group are indicated by black symbols.

The two molecules at the bottom left of Fig. 3 ▸(*b*) feature opposite orientation in [100] and [001] and the same orientation in [010] direction. Their orientations are therefore related by a 2_[010]_ rotation. Observe that when discussing orientation relations of molecules, we only consider the linear part of the operations relating the molecules (the ‘point operation’). An additional translational component of ½**b** means that the molecules are related by a 2_1_ screw rotation as indicated by the blue symbol in Fig. 3 ▸(*b*), whereby the blue color indicates that the operation is not a space group operation of the *Pna*2_1_ polytype.

Double application of this screw rotation gives a **b** translation, which means that the 2_1_ screw rotation is a symmetry element of a layer formed by two half-layers. Combination of the 2_1_ screw rotation and the *a*_[010]_ glide reflection results in inversions, as indicated by blue circles in Fig. 3 ▸(*b*). Pairs of molecules related by inversion correspond to the hydrogen-bond connected pair in Fig. 2 ▸. Combining these symmetries, the overall layer group symmetry is *p*12_1_/*a*1.

Additional POs are identified by considering pairs of red (or yellow) arrows at the center of Fig. 3 ▸(*b*). Here, the orientations are related by an *m*_[001]_ reflection. The intrinsic translational component of the operation relating the molecules is *ca* ¼**a**, resulting in a *glide reflection*. In the OD literature, a generalization of Hermann Mauguin symbols is used and if the translational component were precisely ¼**a**, the glide reflection would be written as *a*_½_. Owing to a small deviation, it is instead written as *a*_*r*_, indicating an intrinsic translation of 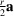. Observe that reflections are the composition of a twofold rotation and an inversion and therefore the subscript *r* is interpreted as in the case of a twofold screw rotation. *r* is a metric parameter of the structure (see below). Double application of the *a*_*r*_ operation results in a translation by *r***a** ≈ ½**a**, which is *not* a (pseudo) translation of the crystal lattice (the lattice formed by all translations of the crystal). Thus, this glide reflection is *not* a symmetry operation of a layer, since it does not map a potential layer onto itself. However it plays a crucial role in the OD description, as it relates two distinct layers. By composition with the *a*_[010]_ operation of the *Pna*2_1_ space group, one obtains a screw rotation with intrinsic translation *ca*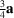 in the [100] direction, with the symbol 2_*r*+1_. Both operations are indicated in Fig. 3 ▸(*b*) by the graphical symbols for regular *a* and 2_1_ operations.

In summary, the structure of **1** can be considered as a structure of layers parallel to (001) with *p*12_1_/*a*1 symmetry. The layer boundaries are indicated in Fig. 3 ▸(*c*). These layers corresponds to hydrogen bonded sheets described in the previous section, though this is purely coincidental. OD layer boundaries may even ‘slice’ through molecules as for example described by Stöger *et al.* (2013 ▸).

Note that the layer boundary is not a plane. The crucial point from an OD perspective is that the layers possess a substantial thickness at every point so that there are only weak interactions across a full layer width. The layers will be designated as *A*_*j*_, *j* being a sequential integer.

### 3.3. OD groupoid family

In a proper OD structure, the layers may contact in different, yet geometrical equivalent ways, resulting in an infinity of stacking possibilities built according to the same symmetry principle. The set of these stacking arrangements form an OD family. The full symmetry of each family member is given by the POs mapping individual layers. These do not form a group, but a groupoid, because an operation that maps layer *A*_*j*_ onto layer *A*_*k*_ can only be composed with an operation that maps layer *A*_*k*_ onto a layer *A*_*l*_ (Ito & Sadanaga, 1976 ▸).

To classify the symmetry of OD families, the groupoids are grouped into OD groupoid families which, in analogy to space group types, abstract from metric parameters (Fichtner, 1977*b* ▸). For the special case of OD structures of one kind of layer with all the same translation lattice there are 400 kinds of OD groupoid families (Fichtner, 1977*a* ▸). The OD groupoid family is derived from the layer symmetry, called λ-POs in the OD literature (λ for layer), and one operation that relates two adjacent layers, called σ-PO (σ for space). The full set of σ-POs is generated by composing the λ-POs with the σ-PO (in mathematical terms, this can be regarded as forming a coset of the layer group). To do so, we use a computer program that we plan to publish in the foreseeable future.

The layer symmetry and a σ-PO were derived in the prior section, leading to the OD groupoid family
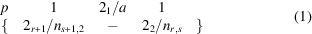
according to a notation inspired by Dornberger-Schiff & Grell-Niemann (1961 ▸). The first line indicates the layer symmetry, the second line the σ-POs using the usual positional Hermann–Mauguin symbols. The meaning of the individual symbols is given in Table 2 ▸, where **c**_0_ is the vector perpendicular to the layer planes with the length of one layer width. *r* and *s* are metric parameters. As noted above, the subscripts of the glide reflections symbols are based on those of twofold rotations. In particular, *n*_*r*,*s*_ in [001] direction standing for a glide with intrinsic translation 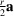 + 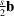 is an unfortunate quirk of the notation, because *n* is defined as a glide reflection with intrinsic translation half the diagonal of a base of the unit cell. The intrinsic translations of symmetry group operations are restricted because *n*-fold application of an operation with linear part of order *n* must result in a lattice translation. In particular, the intrinsic translations of glide reflections and twofold screw rotations (higher order screw rotations cannot exist in layers) must be a full or half of a lattice vector. In contrast, the intrinsic translation of σ-POs is not restricted and, therefore, *r* and *s* may adopt any value, integral, rational or irrational. As will be shown below, these parameters and their deviations from rational values with low denominators have profound implications on the observed diffraction patterns.

### 3.4. Possible layer arrangements and metric parameters

As noted before, the OD groupoid family abstracts from the metric parameters, namely the layer’s cell parameters (*a* = |**a**|, *b* = |**b**|), the layer spacing (*c*_0_ = |**c**_0_|) and the relative positions of adjacent layers with respect to **a** and **b** (*r*, *s*). The latter determine the number of distinct ways in which adjacent layers can be placed and they therefore need to be determined.

There are two ways of generating an ambiguity in the stacking arrangement. Firstly, application of a symmetry operation of layer *A*_*j*_ that does not invert the orientation of **c**_0_ and is not a symmetry operation of *A*_*j*+1_ to the layer pair (*A*_*j*_, *A*_*j*+1_) generates a a new pair (*A*_*j*_, *A*′_*j*+1_), which has the same interatomic distances as the original pair. In **1**, *p*1*a*1 is the subgroup of operations of *A*_*j*_ that does not invert **c**_0_ (called λ-τ-POs in the OD literature). The 2_1_ operation is ignored, as it inverts the orientation of **c**_0_ and therefore maps *A*_*j*+1_ onto the space occupied by *A*_*j*−1_. It consequently does not generate a new (*A*_*j*_, *A*′_*j*+1_) pair. For general *s*, the *a*-glide planes of *A*_*j*_ and *A*_*j*+1_ do not overlap and only the operations of the subgroup *p*1 < *p*1*a*1 also apply to *A*_*j*+1_. Given *A*_*j*_, there are thus [*p*1*a*1:*p*1] = 2 possible ways of placing *A*_*j*+1_ (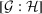 is the index of the subgroup 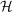 in 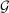).

Secondly, if there is a σ-PO that inverts **c**_0_ (called σ-ρ-PO in the OD literature) and the pair (*A*_*j*_, *A*_*j*+1_) is polar, *i.e.* none of the σ-ρ-POs map *A*_*j*_ onto *A*_*j*+1_*andA*_*j*+1_ onto *A*_*j*_, then application of the inverse of a σ-ρ-PO creates a new pair (*A*_*j*_, *A*′_*j*+1_): *A*_*j*+1_ is mapped onto *A*_*j*_, but *A*_*j*_ is not mapped onto any *A*_*j*+1_ derived by coset decomposition above. Thus the number of stacking possibilities is doubled. In **1**, for general *r*, the 2_*r*+1_ and *n*_*r*,*s*_ operations are of this kind and therefore given *A*_*j*_, there are *Z* = 2[*p*1*a*1:*p*1] = 4 ways of placing the adjacent layers. This is known as the NFZ relationship in the OD literature (Ďurovič, 1997 ▸).

For special values of *r* and *s*, the number of stacking possibilities decreases. Again, we use custom software routines, which we plan to publish in due course, to enumerate these special cases. For the OD groupoid family of **1**, there are the following sets of special parameters:

(i) *s* = *n*, 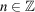: *Z* = 2[*p*1*a*1:*p*1*a*1] = 2

(ii) *r* = *n*, 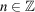: *Z* = [*p*1*a*1:*p*1] = 2

(iii) *r* = *n*, *s* = *m*, 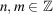: *Z* = [*p*1*a*1:*p*1*a*1] = 1

In the first case, the *a* glide planes of adjacent layers overlap. In the second case, the 2_*r*+1_ operation becomes a symmetry operation of the (*A*_*j*_, *A*_*j*+1_) layer pair, which therefore ceases being polar. In the third case, both conditions are true and the structure is fully ordered, *i.e.* there is no stacking ambiguity. Its space group depends on the parity of *r* and *s*.

Since the major polytype of **1** possesses *Pna*2_1_ symmetry, the *a* glide planes of adjacent layers overlap and we can conclude that *s* is an integer. For even *s*, adjacent *A* layers are related by an *a*_[001]_, for odd *s*, by a *n*_[001]_ glide reflection. In **1**, the former is the case and we conclude that *s* = 0. This can be expressed by the (nonstandard) symbol
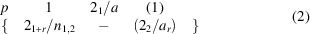
The remaining metric parameter *r* can be derived from the refined coordinates of the **1** molecules. Averaging the atomic coordinates (excluding H atoms) of two independent molecules gives the inversion center of an *A*_*j*_ layer, from which *r* can be inferred. We obtain a clearly non-integral value of *r* = 0.510 which means that the intrinsic translational component of the *a*_*r*_ glide is 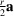 = 0.255**a**.

Thus, given a layer *A*_*j*_, the adjacent layer *A*_*j*+1_ can be placed in two ways, namely application of an *a*_*r*_ or an *a*_−*r*_ operation.

### 3.5. Maximum degree of order (MDO) polytypes

The stacking ambiguity leads to an infinite number of potential polytypes, which are all locally equivalent. Pairs of adjacent layers are always equivalent. However, there are two kinds of triples (*A*_*j*_, *A*_*j*+1_, *A*_*j*+2_): application of twice *a*_*r*_ (or *a*_−*r*_) and application of *a*_*r*_ followed by *a*_−*r*_ (or *vice versa*).

The two polytypes consisting of only either of the triples are said to be of an MDO (Dornberger-Schiff, 1982 ▸). They are particularly simple in the sense that all other polytypes can be decomposed into fragments of MDO polytypes. Moreover, ordered MDO polytypes need the least amount of contextual information during growth. The two MDO polytypes are:

(i) MDO_1_: *P*2_1_/*a*, **c** = 2**c**_0_ + *r***a**, generated by repeated application of *a*_*r*_ (or *a*_−*r*_ for different orientation).

(ii) MDO_2_: *Pna*2_1_, **c** = 2**c**_0_, generated by alternating application of *a*_*r*_ and *a*_−*r*_.

The MDO_2_ polytype corresponds to the refined *Pna*2_1_ structure, fragments of MDO_1_ were observed by ‘phantom’ molecules and additional diffraction peaks.

The point group of the OD groupoid family (the group generated by the linear part of all POs) is *mmm*. The point groups of the MDO polytypes are proper subgroups of *mmm* and they may therefore crystallize as twins if a stacking fault (a fragment of the other MDO polytype) appears.

The expected twin operations are the operations of the *mmm* point group of the OD groupoid family that do not appear in the point group of the polytype. The orientation states are determined formally by a coset decomposition: for the MDO_1_ (*P*2_1_/*a*) polytype there are [*mmm*:2] = 2 orientation states, related by 2_[100]_, *m*_[100]_, 2_[001]_ or *m*_[001]_. If *r* is idealized to ½, the translation lattice of MDO_1_ would be orthorhombic *B*-centered (*oB*). Since the twin operations are point operations of this lattice (the point group of an *oB* lattice is *mmm*), the twinning would be by *metric merohedry*, which means that the direct and reciprocal lattices of both twin domains coincide perfectly (Grimmer & Nespolo, 2006 ▸). Since *r* deviates from the ideal value, the twinning can be considered as being by *pseudo-merohedry*, with the twin obliquity depending on the deviation from *r* = ½.

For MDO_2_ (*Pna*2_1_), there are [*mmm*:*mm*2] = 2 expected twin domains. This is a twin by inversion and, since the inversion is always a symmetry operation of any lattice, a twin by *merohedry*. It can’t be directly showed in our experiment, since the short wavelength of 0.56 Å was far from the absorption edges of the C and O elements, which means that the deviation from Friedel’s ‘law’ (stating that diffraction patterns are centrosymmetric) is too small to determine the volume fraction of the two twin domains (the Flack parameter). In fact, the estimated standard uncertainty of the Flack parameter was higher than 0.5, if refined.

Given the observed diffuse scattering, which implies an intimate stochastic mix of MDO_1_ and MDO_2_ fragments, one can assume that the MDO_2_ fragments appear in both orientations to equal fractions, as per the law of big numbers. The structure refinement was therefore performed with a fixed 1:1 volume ratio.

### 3.6. Other polytypes

Potentially, there are an infinity of polytypes, which can be built of fragments of MDO_1_ and MDO_2_. In this section an in-depth discussion of these polytypes and their symmetries is given. Readers not interested in these details may proceed to §3.7.

We will designate as a *translational period* of a polytype the smallest *n*-tuple of adjacent layers from which the polytype can be constructed by translation. It must contain an even number of layers because *A*_*j*_ with even and odd *j* possess different orientations with respect to [100].

Since two adjacent layers are related either by *a*_+*r*_ or *a*_−*r*_ σ-POs, a classical approach of enumerating polytypes would be generating bi-infinite periodic strings over the binary alphabet {+, −}. Symmetry equivalents (*e.g*. those obtained by switching + and −) are then removed. We will call these strings σ-sequences, as they list the σ-POs. The primitive period of a periodic bi-infinite word is the smallest fragment from which the word can be generated by repeated juxtaposition. Even- length primitive periods correspond to a translation period. For odd lengths, the primitive period must be doubled to obtain a translation period.

Here we propose a different approach, which respects the OD description: consider (periodic) polytypes as a periodic sequence of MDO_1_ and MDO_2_ triples (sharing a layer), represented by a periodic bi-infinite string over the alphabet {1, 2} (the number stands for the kind of MDO triple). These strings will be called stacking sequences. The primitive period of these strings correspond to an asymmetric unit of the polytype and better represents the complexity of a polytype than the σ-sequence. Moreover, weeding out symmetry equivalents is simpler, as only reverse strings have to be removed.

Periodic strings can be enumerated very efficiently according to Duval (1983 ▸), however, for primitive periods of lengths *n* ≥ 6, pairs of non-palindromic reflected strings appear (*e.g.* strings with the primitive period 112122 and 221211), where one instance has to be removed. An efficient algorithm to directly avoid these pairs was described by Sawada (2001 ▸).

The polytypes with stacking sequences up to a primitive period length *n* = 6 are compiled in Table 3 ▸. For odd *n*, the translation period is doubled, owing to the different orientations of the *A*_*j*_ and *A*_*j+n*_ layers. For even *n* with an odd number of MDO_2_ triples in the primitive period, the translation period is likewise doubled. The σ-sequence (third column) and **c** vector (fourth column) are trivially derived by observing that every MDO_2_ triple changes direction (*a*_+*r*_ ↔ *a*_−*r*_).

Since the *a*_[010]_ glide reflection applies to all layers, it is a total operation of every polytype, *i.e* it is element of the symmetry group of the polytype. *Periodic* polytypes possess space group symmetry and their symmetry must therefore be a supergroup of a space group of type *Pa*. Other total symmetry operations can either derive from layer operations (λ-POs) or operations relating layers (σ-POs). A polytype is *P*2_1_/*a* symmetric, if the 2_1_ operation of a layer is a global operation. This is only possible inside MDO_1_ fragments and the remaining part of the primitive period must be palindromic. Thus, to check for *P*2_1_/*a* symmetry, locate all odd-length 1…1 subsequences in the primitive period of the stacking sequence and check if the rest is palindromic. An example is the primitive period 111121112, where 111 is a block of an odd number of MDO_1_ fragments and the remainder 211112 is palindromic.^1^

A polytype is *Pna*2_1_ symmetric, if all layers *A*_*j*_, *A*_*j*+*k*_, where *k* is half the translation period, are related by an *n*_[100]_ glide reflection. This is only possible if *k* is odd (because only then *A*_*j*_ and *A*_*j*+*k*_ have opposite orientation with respect to [100]), which means that the stacking sequence has an odd primitive period. In this case it is sufficient that there is an odd number of MDO_2_ triples in the stacking sequence, because then the second half of the primitive period of the σ-sequence is precisely the reflected version of the first half. Consider for example the stacking sequence with the primitive period 12122 resulting in the σ-sequence with primitive period + + − − + − − + + −. Note that this condition precludes the conditions for *P*2_1_/*a* symmetry, because for odd *n* the latter requires and even number of MDO_2_ triples in the palindromic part of even length.

All other polytypes possess only *Pa* symmetry. It is interesting to investigate the asymptotic behavior for stacking sequences with large periods. The number of polytypes whose stacking sequence has a primitive period of length *n* corresponds to the integer sequence A001371 of the *On-line Encyclopedia of Integer Sequences* (OEIS Foundation Inc., 2025 ▸). Those with *P*2_1_/*a* symmetry are given by the sequence A056513. In the *n* → ∞ limit, the density of *P*2_1_/*a* sequences approaches zero. In contrast, *Pna*2_1_ polytypes appear for precisely half of the stacking sequences with odd periods. Thus, in the limit, 25% of stacking sequences are *Pna*2_1_ and the remaining 75% are *Pa*, whereas for small *n*, *P*2_1_/*a* dominates. Table 4 ▸ lists the numbers for *n* ≤ 30.

### 3.7. Diffraction pattern

**Figure 4:**
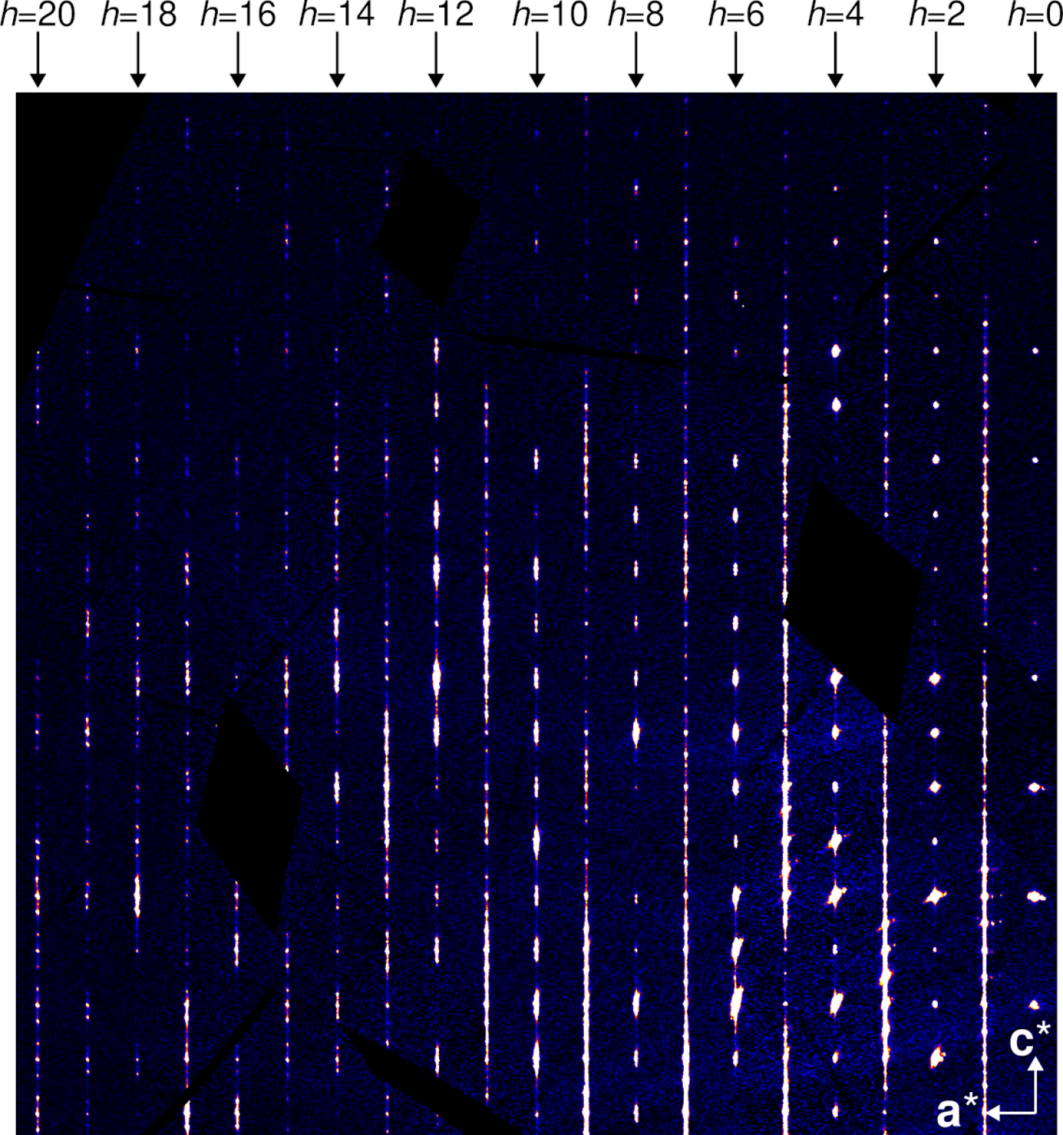
Reconstruction of the *k* = 2 plane from diffraction data. *k* = 2 was chosen arbitrarily. Other *k* constant sections are qualitatively similar, except *k* = 0, which is affected by the systematic absences of the *a* glide reflection common to all polytypes.

The diffraction pattern of **1** is complex, featuring more or less diffuse peaks that can be attributed to different domains on top of pronounced diffuse scattering (Fig. 4 ▸).

Describing the diffraction pattern requires a reciprocal basis (a*, b*, c*)^*T*^ (the superscript *T* stays for the transpose operation, as reciprocal bases are written by convention as columns). Since any periodic polytype must possess a multiple of two layers in the primitive translation period (see above), we will use the dual basis of (**a**, **b**, 2**c**_0_). Since **a**, **b** and **c**_0_ are mutually orthogonal, a* is the vector parallel to **a** with the length 1/*a*, *etc*.

The coordinate triples in reciprocal space will be designated as *hk*ν (**s** = *h*a* + *k*b* + νc*) to highlight that diffraction can occur anywhere on rods parallel to c* with integral *h* and *k*.

A crucial point in understanding diffraction patterns of polytypes is identifying reflections that are common to all domains. Thereto, the *family structure* is determined, which is informally an equal overlay of all possible polytypes. The symmetry group of the family structure is generated by extending the POs to global operations. For the OD family of **1**, let us first concentrate on the group of translations. Layers *A*_*j*_ and *A*_*j*+2*n*_ (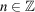) are related by a translation. The vector connecting the origins of both layers can be written as 2*mr***a** + 2*n***c**_0_, −*n* ≤ *m* ≤ *n*, whereby it is useful to reduce the **a** component to its fractional part frac(2*mr*). The metric parameter *r* is not close to a rational with small denominator and can be assumed as being irrational. This means that with growing *n*, frac(2*mr*) can be arbitrarily close to any number in the 0–1 range. Overall, the translation group of the family structure is dense in the **a** direction, and has periodicities **b** and 2**c**_0_ in the other directions. Accordingly, the symmetry of the family structure is *not* a space group, but the smallest supergroup of the space groups of all possible polytypes. Owing to the dense translation vectors in the **a** direction, the **a**-component of glides and screw rotations can likewise be arbitrary small. In the context of Euclidean normalizers (Koch *et al.*, 2016 ▸), similar groups exist, with a technical difference: for normalizers, the set of translations is uncountably infinite, so in a sense the set of translations is even ‘more dense’.^2^ If, nevertheless, the same notation is used, the symmetry group of the family structure can be stated as being of type *P*^1^*bmm* with the ‘basis’ (ɛ**a**, **b**, 2**c**_0_).

While this discussion may appear academical, a crucial consequence is that diffraction intensities may appear anywhere on rods *h* and *k* integral (since any *A*_*j*_ layer can only produce intensities on such rods), except for *h* = 0 rods. There, sharp reflections are observed at positions *k* + *l* even owing to the *b*-glides in the *P*^1^*bmm* group. A statistical stacking without any correlation between layers would produce unstructured diffuse scattering along c* on all rods *h* ≠ 0.

These considerations are only perfectly true if there is no desymmetrization, *i.e.* all layers and all layer contacts are perfectly equivalent. This assumption is confirmed by the diffraction pattern, as indeed for *h* = 0 it features sharp peaks and no diffuse scattering.

### 3.8. Qualitative interpretation of the diffraction pattern

For *h* ≠ 0, diffraction peaks are broader and placed on top of diffuse scattering (Fig. 4 ▸), which means that the crystal is not periodic (or quasi-periodic) and has to be described using disorder models. Nevertheless, it is useful to describe the peak positions qualitatively at first. Peaks corresponding to both MDO polytypes are observed. The reciprocal bases are:

(i) MDO_1_ (first orientation): (a* − *r*c*, b*, c*)^*T*^

(ii) MDO_1_ (second orientation): (a* + *r*c*, − b*, − c*)^*T*^

(iii) MDO_2_ (both orientations): (a*, b*, c*)^*T*^

**Figure 5:**
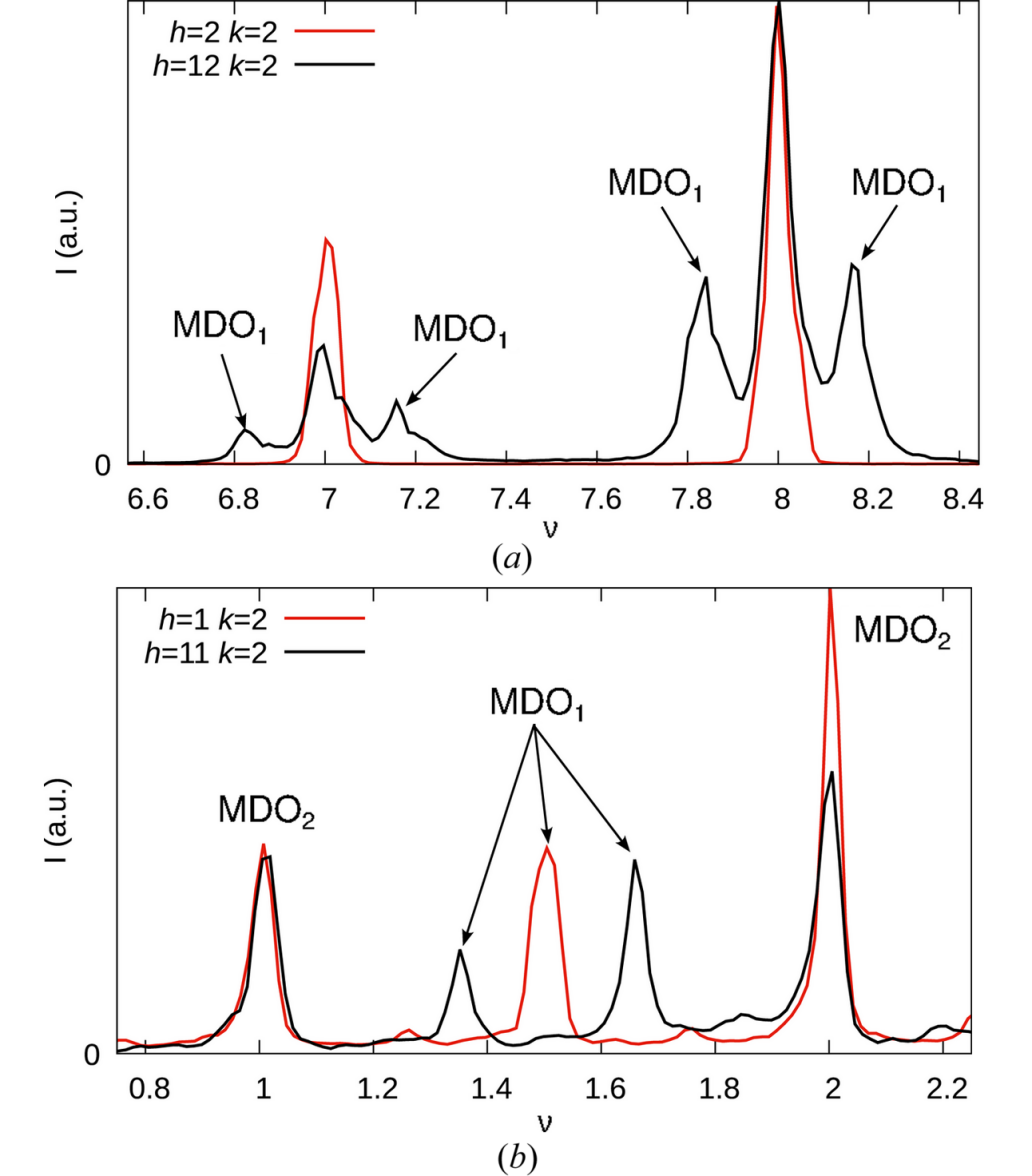
Comparison of experimental reflection profiles on (*a*) even *h* and (*b*) odd *h* rods with low (2, 1) and high (12, 11) *h* values, showing splitting of the MDO_1_ ‘reflections’ for high *h* values resulting in characteristic triple and double peaks, respectively. In all cases, *k* = 2. The high-*h* intensities were scaled by a factor of 3 to bring them to a similar scale as the low-*h* intensities.

The reflections of MDO_2_ are all located at integral ν. Since *r* ≈ ½, for small even |*h*| MDO_1_ reflection are likewise expected close to integral ν. With increasing |*h*|, their positions deviate, resulting in distinct ‘triplets’ of MDO_1_ and MDO_2_ peaks [Fig. 5 ▸(*a*)]. For small odd |*h*|, MDO_1_ reflections are close to half-integer ν. Deviations for increasing |*h*| lead to a distinct splitting into ‘doublets’ [Fig. 5 ▸(*b*)].

**Figure 6:**
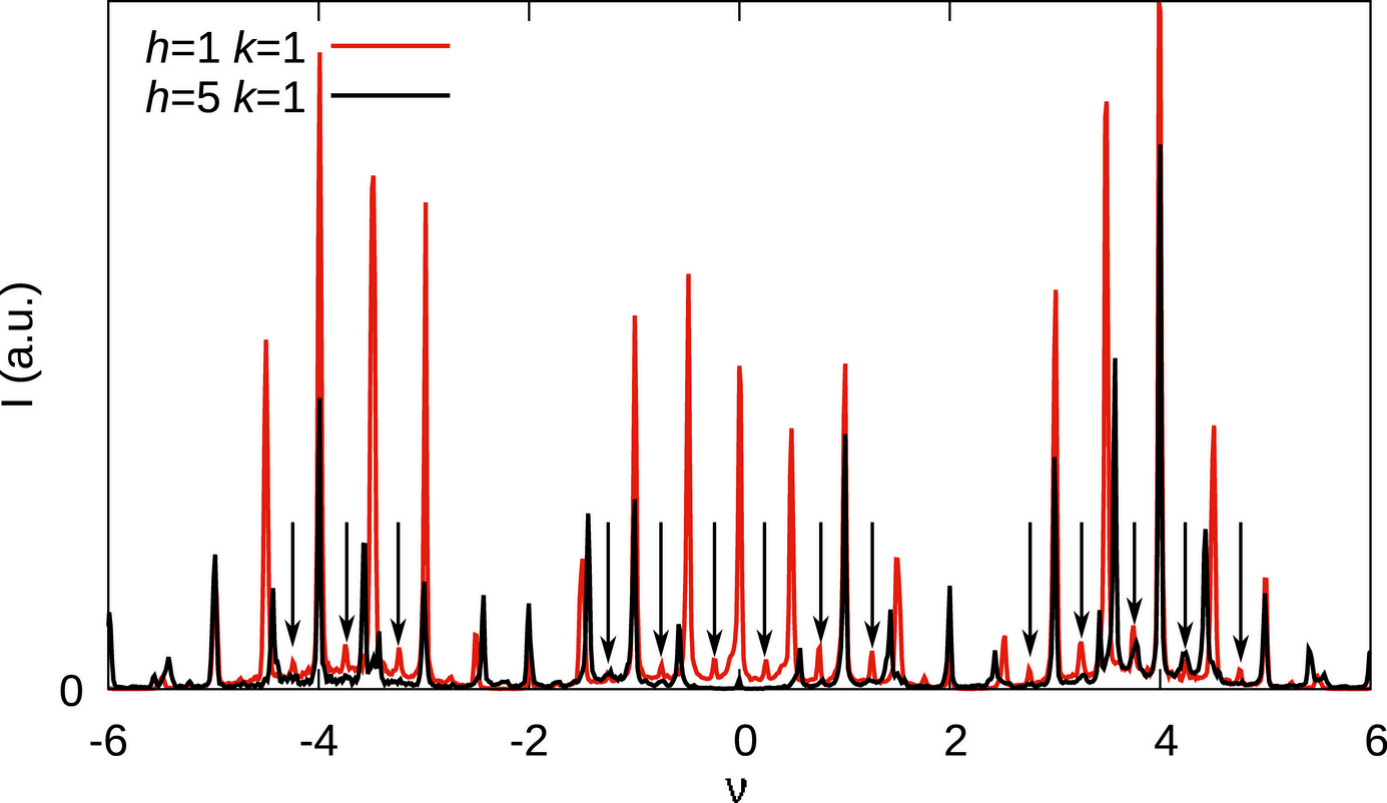
Comparison of reflection profiles on (11ν)* (red) and (51ν)* (black) rods showing additional peaks at quarter points of ν.

Close inspection of the rods with low odd *h* values reveal additional peaks at quarter points of ν, marked by black arrows in Fig. 6 ▸. These peaks are significantly weaker than the MDO_1_/MDO_2_ peaks. For |*h*| = 5 they are barely observed, for |*h*| = 7 they are practically absent. It is unknown whether they belong to distinct domains or are part of the disordered MDO_1_/MDO_2_ domains.

Since there is no broadening, splitting or deviation from 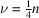 (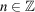) observed up to |*h*| = 5, we assume a polytype with a **c** vector without *r***a** component. The potential polytypes with **c** = 8**c**_0_ are (see §3.6 and Table 3 ▸):

 1112, + + + + − − − −, *P*2_1_/*a*

 1222, + + − + − − + −, *P*2_1_/*a*

 11211222, + + + − − − + −, *Pa*

 11212212, + + + − − + − −, *Pa*

 12122222, + + − − + − + −, *Pa*

Applying Occam’s razor principle we can suppose that the first two, the simplest, polytypes are the most probable. The absence of these peaks on *h* even rods is a case of systematic non-space group absences and is due to *r* ≈ ¼, which are characteristic of OD structures where layers are translationally equivalent. For derivation, see *e.g.*, Ferraris *et al.* (2008 ▸).

If the diffraction peaks are interpreted as Bragg peaks (*i.e.* their shape is only determined by experimental artifacts), then the crystal would be considered as an allotwin composed mostly of MDO_1_ and MDO_2_ with minor domains of an unknown non-MDO polytype, showed by very minor peaks at quarter points of ν, see above. Yet, owing to broad peaks and diffuse scattering, we will categorize the crystal under investigation as an allotwin-like disordered OD polytype. The aforementioned contributions of the non-MDO polytype are so weak that we will ignore them in the upcoming modeling of the diffraction pattern.

### 3.9. Growth models

Here we assume that the crystal is one homogeneous disordered domain, in contrast to distinct independently scattering domains. There are different ways to model such domains. For layer-stacking disorder we find that the most intuitive approach is growth models, which means that the position/orientation of the layer *A*_*j*_ is given in a probabilistic way depending on the *s* prior layers *A*_*j*−*s*_…*A*_*j*−1_, where *s* is called the range of interaction or *reichweite* (Treacy *et al.*, 1991 ▸). It should be stressed that such a description does not describe the actual physico-chemical process of crystal growth, *i.e.* it does not imply that the crystal is grown layer-by-layer. It is merely a mathematical model of expressing probabilities of finding specific layer *n*-tuples. Alternative equivalent descriptions are Ising models (Welberry, 2010 ▸), which have the advantage of being applicable to two-dimensional disorder (*i.e.* arrangements of rods).

The range of interaction *s* is a measure of complexity as is the number of parameters used to describe the model. We will require that the model follows the symmetry of the OD description, because we assume no external influences that could favor one orientation of a layer *n*-tuple over another.

The simplest model is described by a single parameter *p*, the probability of encountering an MDO_1_ fragment (the probability of an MDO_2_ fragment then is 1 − *p*). Expressed as a growth model, it is a range of interaction *s* = 1 model represented by the graph
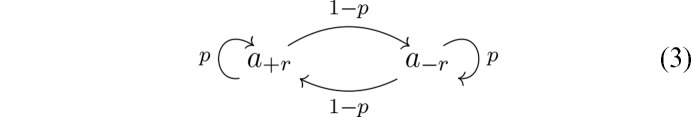
since every step depends only on the prior step. The nodes in the graph represent the σ-PO relating adjacent layers and the edges are marked with the transition probabilities. Strictly speaking, *p* = ½ would represent an *s* = 0 model, as every step is independent. Such a model would result in unstructured diffuse scattering. For *p* > ½ broad peaks at the MDO_1_ positions appear, converging to the Bragg peaks of a 1:1 MDO_1_ twin for *p* → 1. The shape of the peaks is well understood and given for example in Hendricks & Teller (1942 ▸). In return, for *p* → 0 the diffraction pattern converges to the 1:1 MDO_2_ inversion twin. However, there exists no probability *p* for which peaks at the MDO_1_*and* MDO_2_ positions are observed, *i.e.* such a simple model cannot describe the observed diffraction pattern.

The simplest model able to describe an allotwin-like disordered structure possesses two parameters *p*_1_ and *p*_2_ giving the probability of MDO_1_ after MDO_1_ and MDO_2_ after MDO_2_, respectively. It corresponds to the range of interaction *s* = 2 model represented by the graph
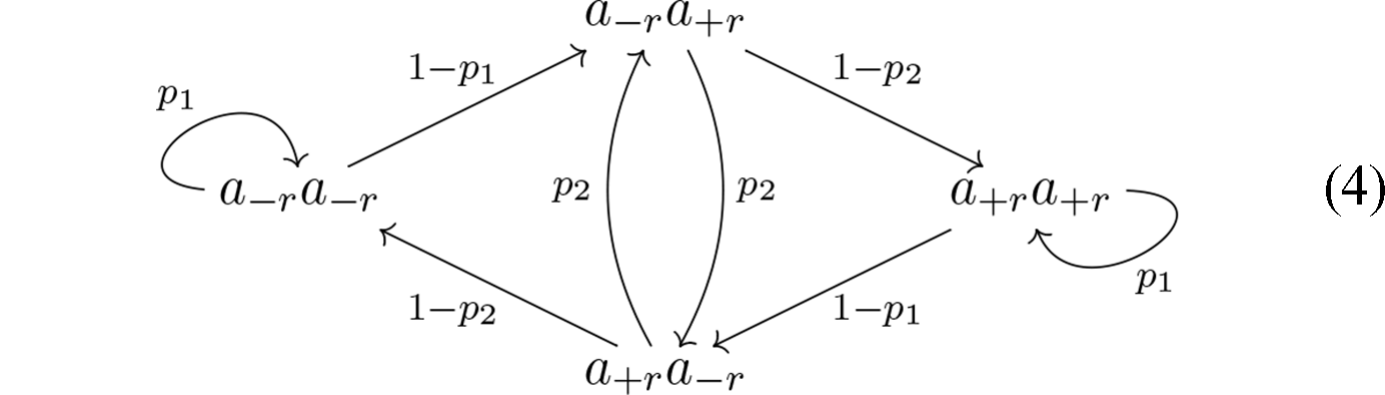
where each node represents the two prior steps. For *p* = *p*_1_ = 1 − *p*_2_ the model degenerates to the simple model of equation (3). The *s* = 2 model has two additional extreme cases:

 *p*_1_, *p*_2_ → 0: the non-MDO polytype with stacking sequence of primitive period 12, respectively σ-sequence of primitive period + + − − + + − − (see §3.6)

 *p*_1_, *p*_2_ → 1: an allotwin of MDO_1_ and MDO_2_

Thus, in a sense an allotwin and the simplest non-MDO polytype possess the same complexity: both require an *s* = 2 range of interaction, in contrast to MDO polytypes (*s* = 1).

Note that only probabilistic statements can be given on such random processes. In particular, in probability theory, statements with a probability *1* should be prefixed with the expression *almost surely*. Here, we will omit this expression for brevity. Solving the steady state of the random process in equation (4) shows that the fraction of MDO_1_ and MDO_2_ triples are 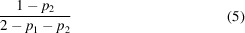
and 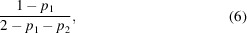
respectively. Another interesting property is the average length of MDO_1_ and MDO_2_ fragments. It is a standard result that these are 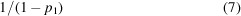
and 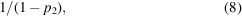
respectively.

### 3.10. Diffuse scattering

Even though modeling diffuse scattering is often considered as being tricky, from a mathematical point of view it is easier to handle than diffraction of periodic structures. The latter is plagued by the issue of sums of regular density functions converging to discrete distributions. The convergence behavior of diffuse scattering is distinctly ‘nicer’ (uniform *versus* distributional convergence) and very reasonable approximations can be obtained with early series terminations. Though general software such as *DIFFaX* (Treacy *et al.*, 1991 ▸) and its derivatives can simulate diffuse scattering of layer structures in the general case, it is instructive to analyze the concrete case of **1** in detail. Readers not interested in structure factor calculations may proceed to §3.11.

Let us assume that the diffraction intensity |*F*|^2^ of a polytype is the sum of the pair correlations between any two pairs of layers (Welberry, 2010 ▸):
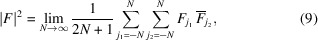
where *F*_*j*_ is the Fourier transform of the layer *A*_*j*_ and an overline indicates the complex conjugate. The overall sum is real, because for every 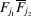 term, it contains the complex conjugate 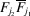. This can be made more explicit by summing over all unordered pairs of layers:
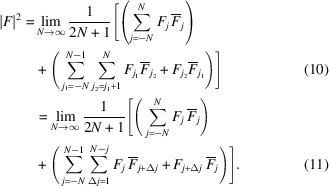
Here, all terms are real (though potentially negative). The second line conveniently groups pair-correlations of layers spaced by Δ*j*.

Note that all even *j* and all odd *j* layers are translationally equivalent and call *F*^+^ and *F*^−^ the Fourier transforms of the respective layers when placed at the origin [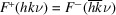]. Then we have: 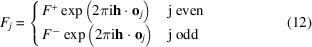
where **o**_*j*_ is the origin of the layer *A*_*j*_ and the terms in equation (16) calculate as 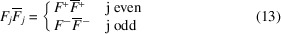
and 
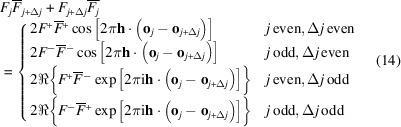
where 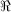 designates the real value.

It is easy to construct layer sequences where the limit of equation (9) does not converge. However stochastic processes such as the one given in equation (4) typically possess very ‘nice’ (exponential) convergence behavior. The expected densities of **o**_*j*_ − **o**_*j*+Δ*j*_ are well defined and easily calculated.

For a given Δ*j* ≥ 1, **o**_*j*_ − **o**_*j*+Δ*j*_ may adopt the form *mr***a**/2 + Δ*j***c**_0_ with *m* being of the same parity as Δ*j* and in the range − Δ*j* → Δ*j*. In the model of equation (9), the expected density of **o**_*j*_ − **o**_*j*+Δ*j*_ is independent of the parity of *j* and will be designated as *P*_Δ*j*_(*m*), which accordingly must fulfill
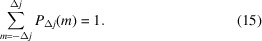
To clarify, let us consider a few examples:

(i) In the MDO_1_ polytype (repeated application of *a*_*r*_), the origins of layers *L*_*j*_ and *L*_*j*+Δ*j*_ are connected by Δ*jr***a**/2 + Δ*j***c**_0_. Thus, *P*_Δ*j*_(*m*) = 1 if *m* = Δ*j* and 0 otherwise.

(ii) In the other orientation of the MDO_1_ polytype (repeated application of *a*_−*r*_), *P*_Δ*j*_(*m*) = 1 if *m* = −Δ*j* and 0 otherwise.

(iii) In a twin of both MDO_1_ orientations with twin volume fraction 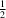, *P*_0_(0) = 1 and for Δ*j* ≠ 0: 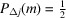 if |*m*| = |Δ*j*| and 0 otherwise.

(iv) In the MDO_2_ polytype (alternating application of *a*_*r*_ and *a*_−*r*_), *P*_Δ*j*_(0) = 1 for even Δ*j* and 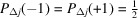 for odd Δ*j*. *P*_Δ*j*_(*m*) = 0 for all other combinations. The same is true for twins of both MDO_2_ orientations, because, by definition, the twin interfaces are infinitely sparse compared to the bulk of the twin domains.

(v) In a purely random stacking, where *a*_*r*_ and *a*_−*r*_ are chosen for each layer independently with a probability of 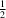, for Δ*j* and *m* of same parity, 
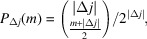
where 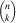 is the binomial coefficient, leading to a Pascal’s triangle-like construction.

For a *p*_1_ ≠ 0, 1 and *p*_2_ ≠ 0, 1 model, the expected densities of the vectors connecting the origins of the layers *L*_*j*_ and *L*_*j*+Δ*j*_ are independent of the parity of *j*. Thus, for a given *mr***a**/2 + Δ*j***c**_0_, the terms in the top two and bottom two cases of equation (14) possess the same expected density of *P*_Δ*j*_(*m*)/2 and we can further simplify the Δ*j* odd case to
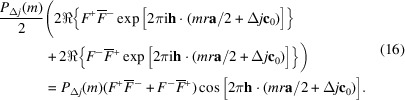
We can now resolve the limits of equation (11). The auto-correlation of layers is 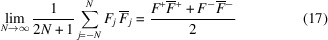
and the cross-correlation of layers spaced by Δ*j*:
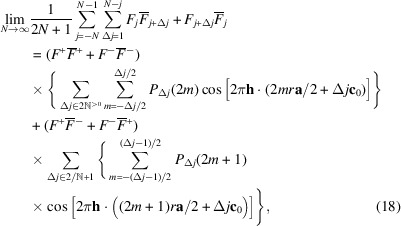
where 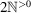 and 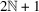 are the sets of positive even and odd numbers, respectively. The scalar product **h** · (*mr***a**/2 + Δ*j***c**_0_) can be written in *hk*ν coordinates as (*mrh* + Δ*j*ν)/2 and thus the diffraction intensity at *hk*ν is given as
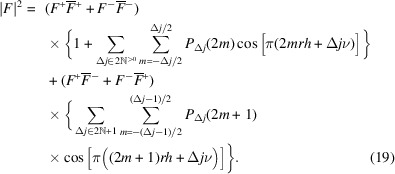
Note that the factors 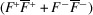 and 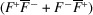 are real and can be pre-calculated for every *h*, *k* rod with the experimental resolution of ν. The *P*_Δ*j*_(*m*) are easily calculated by step-wise development of the stochastic process with the starting probabilities derived from equations (5, 6), For *p*_1_, *p*_2_ not close to 0 or 1, the *P*_Δ*j*_ converge quickly and the series in equation (19) can be truncated at low Δ*j* values. In our simulations, we used Δ*j*_max_ = 40 for adequate results with very short calculation times in the microsecond range.

### 3.11. Properties of the diffuse scattering

**Figure 7:**
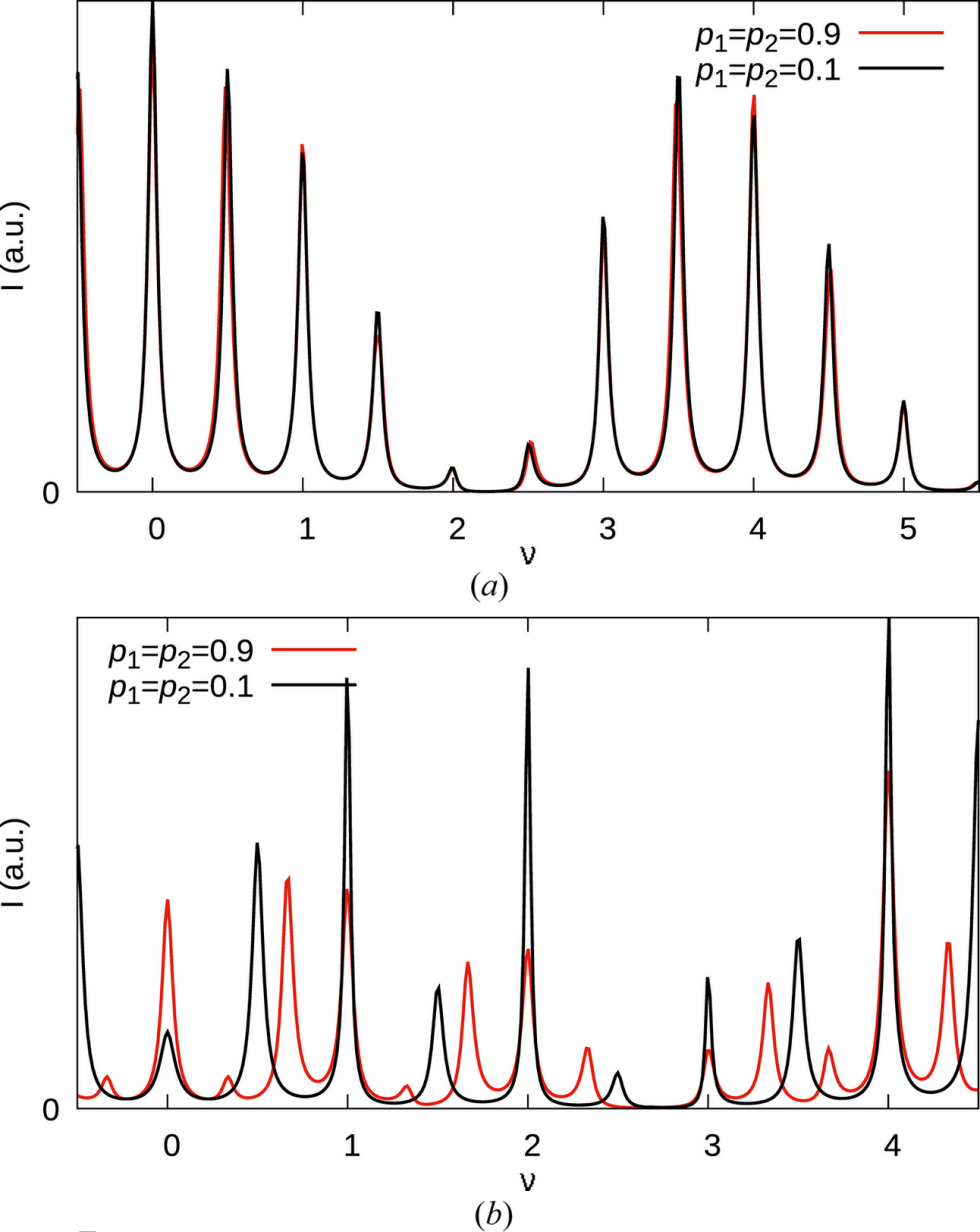
Simulations of the (*a*) (11ν)* and (*b*) 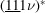 rods with the parameters *p*_1_ =*p*_2_ = 0.9 (red) and *p*_1_ = *p*_2_ = 0.1 (black) and *r* = 0.51.

Before analyzing the actual diffraction pattern, let us examine a few of its properties. When analyzing diffuse scattering, homometry has to be taken into account (Welberry, 2010 ▸), that is distinct models resulting in the same diffraction pattern. As we will show in an upcoming publication, for *r* = ½, there are homometric pairs, in particular models with *p*_1_ = *p*_2_ = *p* and *p*_1_ = *p*_2_ = 1 − *p* describe distinct structures resulting in the same diffraction pattern. Here, this affects only rods with low |*h*| values, because the *hr* factors in equation (19) are close to ½. Consider for example the allotwin-like *p*_1_ = *p*_2_ = 0.9 and the non-MDO-like *p*_1_ = *p*_2_ = 0.1 structures. Both are composed of an equal amount of MDO_1_ and MDO_2_ triples. However in the former, the average length of MDO_1_ and MDO_2_ fragments is 10, in the latter 10/9 ≈ 1.11, *i.e.* the structures are fundamentally different. Yet, the *h* = 1 rods of both structures are practically indistinguishable [Fig. 7 ▸(*a*)]. There are only very subtle changes in the peak heights and positions. In contrast, the *h* = 11 rods differ substantially [Fig. 7 ▸(*b*)]. In particular, the peaks at half-integer ν are split in the allotwin-like phase, but remain unsplit in the non-MDO-like phase, as expected. Note that homometric mates also exist for pairs *p*_1_ ≠ *p*_2_ in the case of *r* = ½, though the deviation of their growth-model probabilities is non-trivial and will be discussed in a future publication. In these cases too, differences in the diffraction pattern increase with |*h*|.

Thus, in the present case, it is most productive to analyze rods with high *h* values. Moreover, the splitting of the peaks in the experimental data [Fig 5 ▸(*b*)] clearly shows that only models with *p*_1_, *p*_2_ > ½ have to be considered.

**Figure 8:**
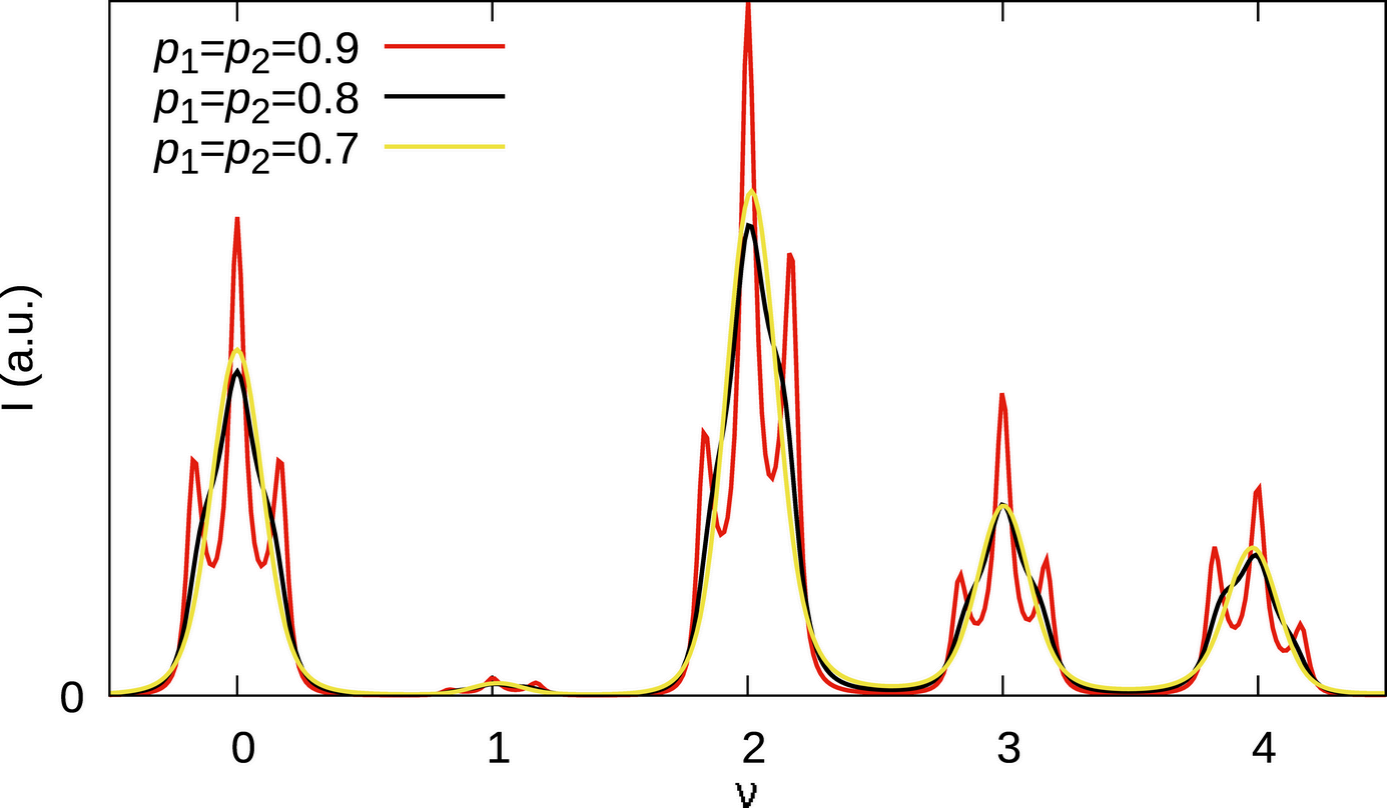
Simulations of the 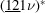 rod with the parameters *p*_1_ = *p*_2_ = 0.9 (red), *p*_1_ = *p*_2_ = 0.8 (black) and *p*_1_ = *p*_2_ = 0.7 (yellow) and *r* = 0.51. With decreasing *h*, the broadening / splitting is even less pronounced.

Let us further evaluate the effect of the parameters *p*_1_, *p*_2_ on characteristic triplet peaks at high even *h* values [see experimental data in Fig. 5 ▸(*b*)]. Fig. 8 ▸ shows the 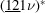 rod for *p*_1_ = *p*_2_ = 0.9, 0.8, 0.7 models. These correspond to allotwin-like disordered structures with equal amounts of MDO_1_ and MDO_2_ triples and average MDO_1_ and MDO_2_ fragment sizes of 10, 5 and 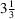, respectively. A clear separation of MDO_1_ and MDO_2_ peaks is only observed for the *p*_1_ = *p*_2_ = 0.9 case. For *p*_1_ = *p*_2_ = 0.8 there is only a single peak with slight shoulders. A qualitative interpretation in this case would suggest an MDO_2_ disordered phase, even though the structure is built of equal amounts of MDO_1_ and MDO_2_ fragments, which proves that such interpretations are treacherous. The diffraction intensities of the *p*_1_ = *p*_2_ = 0.8 and *p*_1_ = *p*_2_ = 0.7 models are barely distinguishable though the average size of the fragments decreased by 33%. This shows that determining the actual domain size is very difficult in highly disordered polytypes. Given the clear splitting into triplets in the experimental pattern [compare Fig. 5 ▸(*b*)] one can expect values of *p*_1_ and *p*_2_ close to 1.

### 3.12. Semi-quantitative interpretation of the diffuse scattering

Despite the expected difficulties mentioned in the last section, here we will try to give a semi-quantitative evaluation of the diffuse scattering.

**Figure 9:**
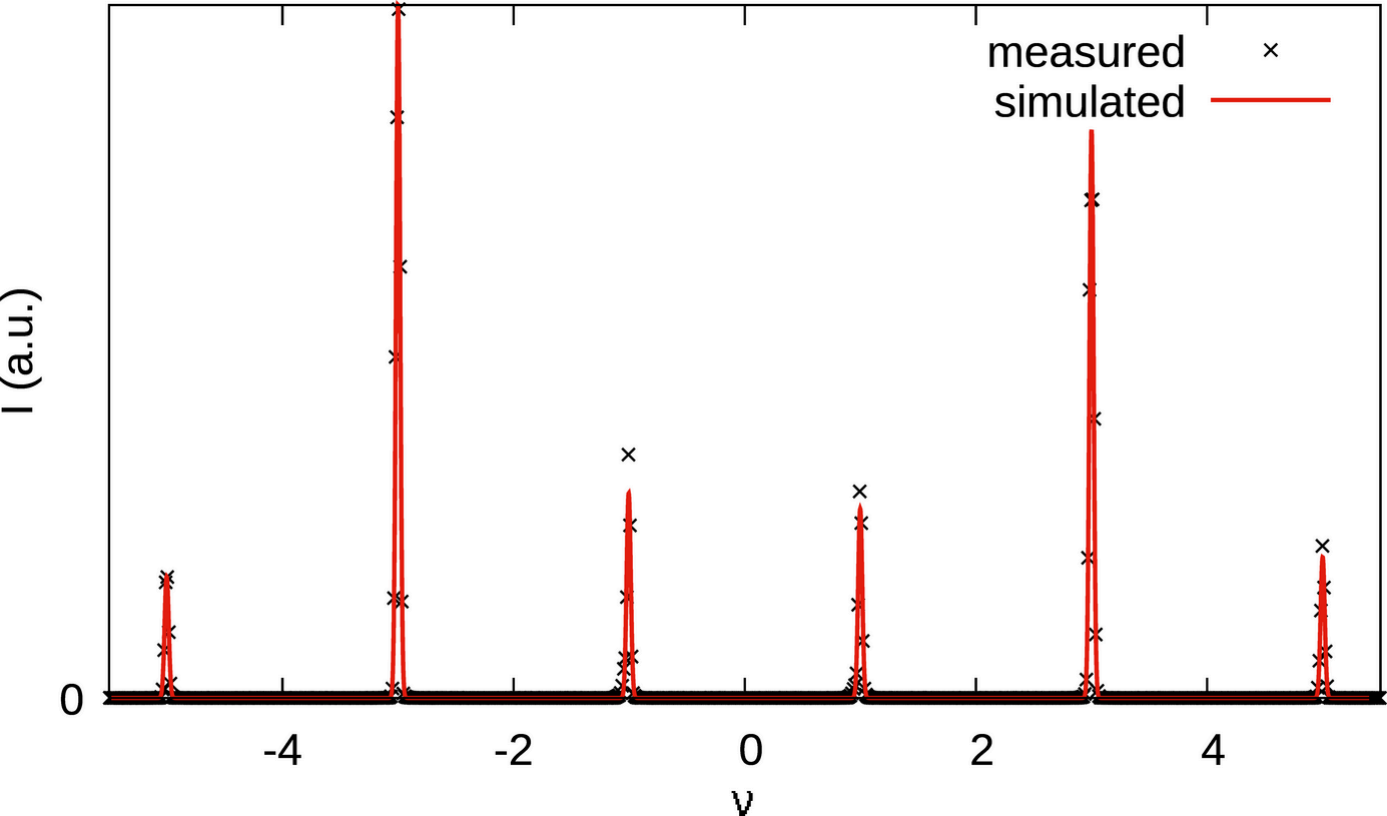
Experimental profile on the (01ν)* rod (black crosses) and fitted sum of normal distributions (red line) with common variance.

One issue to consider is that the shape of the diffuse scattering is convolved with an experimental profile. Even though we minimized artifacts by using synchrotron radiation featuring high monochromaticity and low beam divergence, some broadening due to still not fully perfect beam, detector resolution, and most notably crystal size and mosaicity cannot be prevented. Moreover, the probabilities *p*_1_, *p*_2_ may change throughout the macroscopic crystal. As noted in §3.7, the *h* = 0 peaks can be considered being mostly unaffected by diffuse scattering. Therefore we used these sharp peaks to estimate the experimental broadening. By fitting a sum of normal distributions on the peaks 01*l*, *l* = −5…5 extracted from two-dimensional reconstructed reciprocal space sections, we obtained a variance of σ^2^ = 1.32352 (12) in pixels (Fig. 9 ▸). The peak profile is well described by a Gauss function. A small anisotropy at the base of some peaks is due to parasitic diffraction of additional domains, respectively mosaicity. By using high quality synchrotron radiation, the broadening is tiny, but as we will show below not negligible.

We optimized the model based on two sections of two high-*h* rods (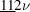 and 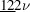) using the MCS algorithm (Huyer & Neumaier, 1999 ▸), which performs a global search. In contrast to classical local searches, a global search finds all local minima, which might be necessary in the case of (pseudo-)homometry. Here, owing to high-*h* values this was not the case.

**Figure 10:**
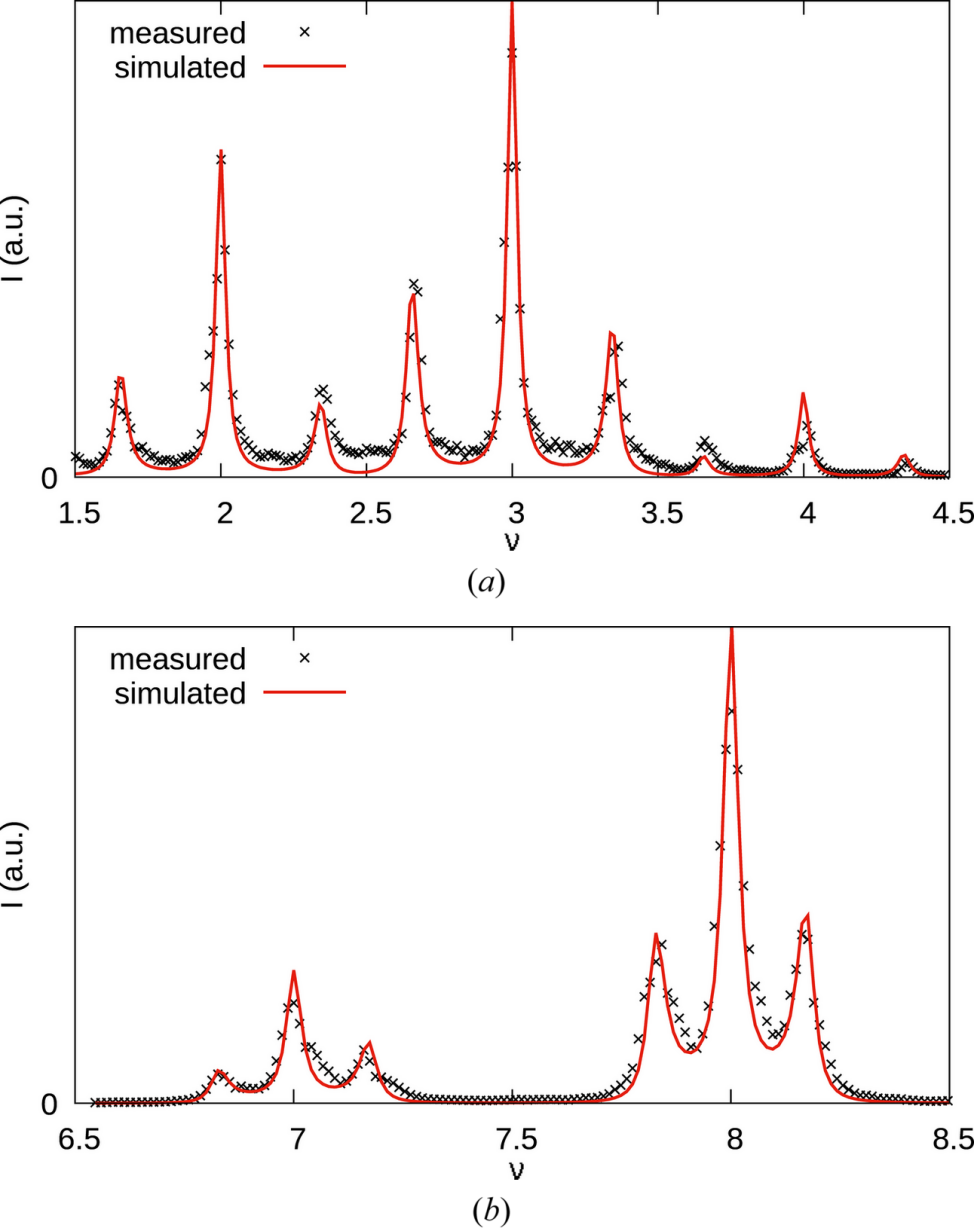
Optimization results on the (*a*) 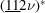 and (*b*) 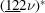 rods. Simulated and measured intensity profiles are given by red lines and black crosses, respectively.

Owing to inconsistencies in the reciprocal space reconstructions, which are due to different crystal positions with different incoherent scattering and absorption effects, we only optimized short sections of the rods. The refined parameters were: center in pixels, length of c* in pixels, *r*, *p*_1_ and *p*_2_. We performed optimizations with and without convolution of the experimental normal distribution. The loss function was the unweighted *R*_p_ (the average difference between calculated and measured intensity, p for profile), as determined by a linear regression. Fig. 10 ▸ shows the measured and simulated intensities of the optimized model. Table 5 ▸ summarizes the refined parameters.

Generally we found simulations of *h* even rods much more robust than of *h* odd rods, which is reflected by significantly improved *R*_p_ values. As an example of a mediocre fit, see for example the valley at ν = 2.5 in Fig. 10 ▸(*a*). In a different structure (publication in preparation) we found these errors to be due to unaccounted desymmetrization: In MDO_1_ fragments and MDO_2_ fragments one can expect the molecules to adopt slightly different orientations. There, better fits of the valleys were obtained by using different structure factors for distinct fragments. Thereto, structures of alternative polytypes have to be determined either experimentally by growing crystals of distinct polytypes or by theoretical calculations, based on quantum-chemistry or molecular mechanics.

Despite these issues, as well as parasitic diffraction resulting in shoulders, the evaluation of the parameters *p*_1_ and *p*_2_ seems to be rather reproducible. They are determined by the width of the peaks, but also the relative heights of MDO_1_*versus* MDO_2_ peaks. When convolving with the experimental profile one finds *p*_1_ ≈ 0.93 and *p*_2_ ≈ 0.94, which would correspond to an allotwin-like structure with *ca* 47% MDO_1_ and 53% MDO_2_ fragments. Though, given the inherent imprecision, one probably should consider the structure as being composed of approximately equal amounts of MDO_1_ and MDO_2_. The average length of the fragments is *ca* 14 (MDO_1_) and 17 (MDO_2_).

By not taking into account the experimental peak broadening, the values of *p*_1_ and *p*_2_ decrease to *p*_1_ ≈ 0.91 and *p*_2_ ≈ 0.92, which proves that even with low-divergence synchrotron radiation the experimental peak profile cannot by neglected completely.

The refined metric parameter *r* ≈ 0.515 is within 1% of *r* derived from the structural refinements (*r* = 0.51034, see §3.4). The latter is certainly the more precise value, though it has to be noted that again *r* may be affected by desymmetrization and change for different local environments in the structure.

## 4. Conclusion and outlook

The title compound is further proof of the fascinating complexity that often arises from polytypism, with a single crystal showing peaks that can be attributed to three distinct polytypes. It also confirms the pervasiveness of OD structures and the generality of OD theory. However, software support such as automatic determination of partial pseudo-symmetry is still lacking and needs to be improved.

Our analysis shows that a quantitative interpretation of the diffraction pattern of disordered crystals can be very treacherous. The occurrence of fragments of minor polytypes are easily underestimated. Pronounced changes in the stacking probabilities result in only mild changes of the peak profiles, which are however affected by experimental artifacts. The robustness of classical single-crystal diffraction is due to the ability of ignoring real structure effects leading to peak broadening by reducing intensities to discrete values and a luxurious data to parameter ratio. Both are lost when analyzing diffuse scattering. On the flip side, crucial structural information is lost by averaging over all unit cells in the crystal, as is done in a classical structure refinement.

Notably, the analysis of the diffuse scattering provides a complexity measure of the structure, namely the range of interaction. To properly describe the observed diffraction pattern, it was necessary to use a range of interaction *s* = 2 model, which allows the modeling of an allotwin-like disordered structure. The same parametrization can describe the simplest of the non-MDO polytype. The crystal under investigation also featured minor amounts of a more complex non-MDO polytype, which requires a longer range of interaction.

## Supplementary Material

Crystal structure: contains datablock(s) I. DOI: 10.1107/S205252062500914X/dk5140sup1.cif

Structure factors: contains datablock(s) I. DOI: 10.1107/S205252062500914X/dk5140Isup2.hkl

CCDC reference: 2496234

## Figures and Tables

**Table 1:** Experimental details

Crystal data	
Chemical formula	C_10_H_12_O_5_
*M*_r_	212.20
Crystal system, space group	Orthorhombic, *Pna*2_1_
Temperature (K)	170
*a*, *b*, *c* (Å)	18.2995 (2), 6.5887 (1), 16.7828 (2)
*V* (Å^3^)	2023.50 (4)
*Z*, *Z*′	8, 2
Radiation type, λ (Å)	Synchrotron, 0.56000
ρ_calc_ (g cm^−3^)	1.393
μ (mm^−1^)	0.07
Crystal shape, color	Block, colorless
Crystal size (mm)	0.21 × 0.14 × 0.06

Data collection	
Diffractometer	Huber Eulerian Cradle
Absorption correction	Multi-scan (*CrysAlis PRO*). Empirical absorption correction using spherical harmonics, implemented in SCALE3 ABSPACK scaling algorithm.
*T*_min_, *T*_max_	0.779, 1.000
No. of measured, independent and observed [*I* > 2σ(*I*)] reflections	66327, 9320, 7252
*R*_int_	0.075
(sin θ/λ)_max_ (Å^−1^)	0.901

Refinement	
*R*[*F*^2^ > 2σ(*F*^2^)], *wR*(*F*^2^), *S*	0.082, 0.255, 1.08
No. of reflections	9320
No. of parameters	386
No. of restraints	11
H-atom treatment	H atoms treated by a mixture of independent and constrained refinement.
Δρ_max_, Δρ_min_ (e Å^−3^)	0.90, −0.32
Absolute structure	Modelled as perfect inversion twin.
Absolute structure parameter	0.5 (fixed)

**Table 2:** Intrinsic translation components of the σ-POs in the OD groupoid symbol of equation (1)

Symbol	Direction	Type	Intrinsic translation
2_*r*+1_	[100]	Screw rotation	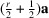
*n*_*s*+1, 2_	[100]	Glide reflection	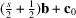
2_2_	[001]	Screw rotation	**c**_0_
*n*_*r*, *s*_	[001]	Glide reflection	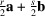

**Table 3:** Polytypes with stacking sequences of primitive period with lengths *n* = 1…6 The stacking sequences and σ-sequences are given by a primitive period.

*n*	Stacking sequence	σ-sequence	**c**_0_	Symmetry
1	1	+ +	2*r***a** + 2**c**_0_	*P*2_1_/*a* (MDO_1_)
1	2	+ −	2**c**_0_	*Pna*2_1_ (MDO_2_)
2	12	+ + − −	4**c**_0_	*P*2_1_/*a*
3	112	+ + + − − −	6**c**_0_	*Pna*2_1_
3	122	+ + −	2*r***a** + 6**c**_0_	*P*2_1_/*a*
4	1112	+ + + + − − − −	8**c**_0_	*P*2_1_/*a*
4	1122	+ + + −	2*r***a** + 4**c**_0_	*Pa*
4	1222	+ + − + − − + −	8**c**_0_	*P*2_1_/*a*
5	11112	+ + + + + − − − − −	10**c**_0_	*Pna*2_1_
5	11122	+ + + + −	6*r***a** + 10**c**_0_	*P*2_1_/*a*
5	11212	+ + + − −	2*r***a** + 10**c**_0_	*P*2_1_/*a*
5	11222	+ + + − + − − − + −	10**c**_0_	*Pna*2_1_
5	12122	+ + − − + − − + + −	10**c**_0_	*Pna*2_1_
5	12222	+ + − + −	2*r***a** + 10**c**_0_	*P*2_1_/*a*
6	111112	+ + + + + + − − − − − −	12**c**_0_	*P*2_1_/*a*
6	111122	+ + + + + −	4*r***a** + 6**c**_0_	*Pa*
6	111212	+ + + + − −	2*r***a** + 6**c**_0_	*P*2_1_/*a*
6	111222	+ + + + − + − − − − + −	12**c**_0_	*P*2_1_/*a*
6	112122	+ + + − − + − − − + + −	12**c**_0_	*Pa*
6	112222	+ + + − + −	2*r***a** + 6**c**_0_	*Pa*
6	121222	+ + − − + −	6**c**_0_	*Pa*
6	122222	+ + − + − + − − + − + −	12**c**_0_	*P*2_1_/*a*

**Table 4:** Number and fractions of polytype symmetries with stacking sequences of primitive period with length *n* = 0…30 The three rightmost columns give the fractions of all stacking sequences of period ≤ *n*.

					Running fractions (%)		
*n*	All	*P*2_1_/*a*	*Pna*2_1_	*Pa*	*P*2_1_/*a*	*Pna*2_1_	*Pa*
1	2	1 (50.0%)	1 (50.0%)	0 (0.0%)	50.0	50.0	0
2	1	1 (100.0%)	0 (0.0%)	0 (0.0%)	66.7	33.3	0
3	2	1 (50.0%)	1 (50.0%)	0 (0.0%)	60.0	40.0	0
4	3	2 (66.7%)	0 (0.0%)	1 (33.3%)	62.5	25.0	12.5
5	6	3 (50.0%)	3 (50.0%)	0 (0.0%)	57.1	35.7	7.1
6	8	4 (50.0%)	0 (0.0%)	4 (50.0%)	54.5	22.7	22.7
7	16	7 (43.8%)	8 (50.0%)	1 (6.2%)	50.0	34.2	15.8
8	24	10 (41.7%)	0 (0.0%)	14 (58.3%)	46.8	21.0	32.3
9	42	14 (33.3%)	21 (50.0%)	7 (16.7%)	41.3	32.7	26.0
10	69	21 (30.4%)	0 (0.0%)	48 (69.6%)	37.0	19.7	43.4
11	124	31 (25.0%)	62 (50.0%)	31 (25.0%)	32.0	32.3	35.7
12	208	42 (20.2%)	0 (0.0%)	166 (79.8%)	27.1	19.0	53.9
13	378	63 (16.7%)	189 (50.0%)	126 (33.3%)	22.7	32.3	45.1
14	668	91 (13.6%)	0 (0.0%)	577 (86.4%)	18.8	18.4	62.9
15	1214	123 (10.1%)	607 (50.0%)	484 (39.9%)	15.0	32.3	52.8
16	2220	184 (8.3%)	0 (0.0%)	2036 (91.7%)	12.0	17.9	70.1
17	4110	255 (6.2%)	2055 (50.0%)	1800 (43.8%)	9.4	32.4	58.2
18	7630	371 (4.9%)	0 (0.0%)	7259 (95.1%)	7.3	17.6	75.1
19	14308	511 (3.6%)	7154 (50.0%)	6643 (46.4%)	5.6	32.5	61.9
20	26931	750 (2.8%)	0 (0.0%)	26181 (97.2%)	4.3	17.4	78.3
21	50944	1015 (2.0%)	25472 (50.0%)	24457 (48.0%)	3.2	32.7	64.1
22	96782	1519 (1.6%)	0 (0.0%)	95263 (98.4%)	2.4	17.3	80.3
23	184408	2047 (1.1%)	92204 (50.0%)	90157 (48.9%)	1.8	32.8	65.4
24	352450	3030 (0.9%)	0 (0.0%)	349420 (99.1%)	1.4	17.2	81.4
25	675180	4092 (0.6%)	337590 (50.0%)	333498 (49.4%)	1.0	32.8	66.2
26	1296477	6111 (0.5%)	0 (0.0%)	1290366 (99.5%)	0.7	17.1	82.1
27	2493680	8176 (0.3%)	1246840 (50.0%)	1238664 (49.7%)	0.5	32.9	66.6
28	4805388	12222 (0.3%)	0 (0.0%)	4793166 (99.7%)	0.4	17.1	82.5
29	9272778	16383 (0.2%)	4636389 (50.0%)	4620006 (49.8%)	0.3	32.9	66.8
30	17919558	24486 (0.1%)	0 (0.0%)	17895072 (99.9%)	0.2	17.1	82.7

**Table 5:** Optimization results with and without convolution with experimental peak profile

	With convolution		Without convolution	
	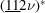	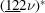	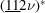	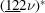
Center (pixels)	1567.68	1570.00	1567.77	1570.00
|c*| (pixels)	74.123	74.854	74.103	74.851
*r*	0.5141	0.5146	0.5140	0.5149
p_1_	0.9273	0.9301	0.9036	0.9052
*p*_2_	0.9428	0.9399	0.9226	0.9173
*R*_*p*_	0.0522	0.0081	0.0389	0.0087

## Footnotes

1 This example was chosen such that the central MDO_1_ block is not at the beginning in the lexicographically sorted representative, which are the strings output from Duval’s algorithm.

2 Here, the translations in [100] direction form a -module, for Euclidean normalizers they are isomorphic to .
